# Impaired myocardial perfusion is associated with extracellular volume expansion, disease activity and impaired strain and strain rate in systemic sclerosis: a cardiovascular magnetic resonance study

**DOI:** 10.1186/1532-429X-17-S1-Q71

**Published:** 2015-02-03

**Authors:** Ntobeko A Ntusi, Emily Sever, Joseph Lockey, Jane M Francis, Stefan K Piechnik, Vanessa M Ferreira, Paul M Matthews, Paul B Wordsworth, Stefan Neubauer, Theodoros D Karamitsos

**Affiliations:** 1 OCMR, Division of Cardiovascular Medicine, University of Oxford, Oxford, UK; 2 Division of Cardiology, Department of Medicine, University of Cape Town, Cape Town, South Africa; 3 Division of Brain Sciences, Imperial College London, London, UK; 4 Bortnar Institute, Nuffield Department of Orthopaedics, Rheumatology and Musculoskeletal Sciences, University of Oxford, Oxford, UK

## Background

Systemic sclerosis (SSc) is characterised by vascular dysfunction and multi-organ fibrosis, with the heart commonly involved. Cardiovascular disease (CVD) in SSc may be direct or indirect, but often remains subclinical. SSc patients with apparent cardiovascular clinical features are at greater risk of deterioration and premature cardiovascular death, often from complications of myocardial ischaemia. CMR first-pass perfusion detects myocardial ischaemia with great accuracy. We hypothesised that CMR first-pass perfusion would be able to differentiate between segmental (indicating epicardial coronary artery disease) and non-segmental subendocardial (indicating microvascular dysfunction) perfusion defects in patients with SSc; and that microvascular dysfunction (relating to chronic myocardial inflammation) was more frequent in SSc.

## Methods

17 SSc patients (16 female, mean age 55 ± 9 years) and 17 matched controls (16 female, mean age 54 ± 10 years) were enrolled. All patients with known cardiovascular disease were excluded. Study participants underwent CMR at 1.5T and the assessments included cine, tagging, T1 mapping, T2-weighted, perfusion, late gadolinium imaging (0.15mmol/kg gadoderic acid - Dotarem®) and ECV quantification. Comorbid status, disease activity index (VDAI score) and duration of disease were recorded for each subject.

## Results

Myocardial perfusion reserve index was 1.5 ± 0.3 and 2.0 ± 0.4 (p<0.001) in SSc and controls, respectively. Non-segmental (circumferential) subendocardial perfusion defects were seen in 41% of SSc and none (p<0.001) of controls studied. There was no significant difference in LV size, mass and ejection fraction between SSc patients and controls. Peak systolic circumferential strain and peak diastolic strain rate were impaired in patients. Impaired MPRI correlated with peak systolic strain (R -0.91, p<0.001) and peak diastolic strain rate (R 0.56, p<0.001) in SSc. Furthermore, abnormal MPRI correlated with VDAI (R -0.58, p=0.02) and ECV (R -0.56, p=0.04) in SSc.

## Conclusions

Myocardial perfusion is impaired in asymptomatic SSc patients with apparently normal hearts. Abnormal perfusion correlates with strain, strain rate, disease activity and ECV in SSc. CMR can detect subclinical cardiovascular involvement in SSc.

## Funding

This study was funded by investigator-led grants from Guerbet and GlaxoSmithKline.

**Table 1:** Baseline characteristics of SSc patients and controls

	Controls N=17	SSc N=17	P value
Female sex, n (%)	16 (94)	16 (94)	1.00

Age, years	55 ± 9	54 ± 10	0.76

Hypertension, n (%)	1 (6)	4 (24)	0.34

Diabetes, n (%)	0	0	-

Hyperlipidaemia, n (%)	2 (12)	3 (18)	0.63

BMI, kg/m2	24 ± 4	27 ± 7	0.21

Methotrexate, n (%)	N/A	5 (29)	-

Chloroquine, n (%)	N/A	1 (6)	-

Leflunomide, n (%)	N/A	1 (6)	-

Prednisolone, n (%)	N/A	1 (6)	-

NSAID, n (%)	N/A	3 (18)	-

HRT/OCP	3 (18)	3 (18)	1.00

mRSS	N/A	16 ± 6	-

VDAI	N/A	4 (2-5)	-

ESR, mm/hr (median, IQR)	N/A	11 (8-15)	-

CRP, mg/L (median, IQR)	1 (1-2)	6 (3-11)	<0.001

Duration of SSc, years (median, IQR)	N/A	13 (7-16)	-

Duration of DMARDs, years (median, IQR)	N/A	4 (2-5)	-

Continuous data are mean ± SD unless otherwise indicated.

Categorical data are frequency (percent) unless otherwise indicated.

BMI, body mass index; CRP, C-reactive protein; DMARD, disease modifying anti-rheumatic drug(s); ESR, erythrocyte sedimentation rate; HRT/OCP, hormone replacement therapy or oral contraceptive pill; IQR, interquartile range; mRSS, modified Rodnan skin score; NSAID, non-steroidal anti-inflammatory drug(s); SSc, systemic sclerosis; VDAI, Valentini disease activity index

**Table 2:** Myocardial structure, function and perfusion in SSc patients and controls

	Controls N=17	SSc N=17	P value
LVEDV indexed to BSA, ml/m2	78 ± 17	74 ± 11	0.15

LVESV indexed to BSA, ml/m2	21 ± 5	18 ± 5	0.06

LVEF, %	74 ± 6	73 ± 5	0.36

LV Mass indexed to BSA, g/m2	51 ± 12	51 ± 8	0.89

LA size, mm	28 ± 5	37 ± 6	<0.001

Mid SA circumferential strain	-18.7 ± 1.0	-17.0 ± 1.7	<0.001

Peak diastolic circumferential strain rate (s-1)	114 ± 16	86 ± 24	<0.001

Presence of LGE (%)	0	10 (59)	-

Volume fraction of LGE>2SD (%)	0	2.6 ± 0.3	-

Global myocardial T2 SI Ratio	1.5 ± 0.2	1.7 ± 0.4	0.08

Volume fraction of oedema by T2 (%)	0	18 (7-23)	-

Average myocardial T1, ms	958 ± 22	1, 008 ± 28	<0.001

Volume fraction of T1>990ms (%)	0	59 (43-71)	-

ECV (%)	27.9 ± 2.4	35.4 ± 4.7	<0.001

Rest RPP	7, 989 ± 1, 280	8, 478 ± 1, 829	0.37

Stress RPP	11, 732 ± 1, 789	11, 980 ± 1, 790	0.69

MPRI	2.0 ± 0.4	1.5 ± 0.3	<0.001

Proportion of non-segmental perfusion defects (%)	0	7 (41)	-

Continuous data are mean ± SD unless otherwise indicated.

ECV, extracellular volume; LA, left atrium; LGE, late gadolinium enhancement; LV, left ventricle/ventricular; LVEDV, left ventricular end-diastolic volume; LVEF, left ventricular ejection fraction; LVESV, left ventricular end-systolic volume; MPRI, myocardial perfusion reserve index; RPP, rate pressure product; SA, short axis; SI, signal intensity, SSc, systemic sclerosis

